# Inflammatory Bowel Disease in a Child with Sickle Cell Anemia

**DOI:** 10.1155/2014/732785

**Published:** 2014-06-29

**Authors:** Khaled Alqoaer, Mohammed M. Ahmed, Efteraj S. Alhowaiti

**Affiliations:** Pediatric Department, Prince Salman North West Armed Forces Hospital, P.O. Box 100, Tabuk 71411, Saudi Arabia

## Abstract

Sickle cell anemia (SCA) is a chronic haemoglobinopathy that can affect many organs in the body including gastrointestinal tract. However, colonic involvement is very rare and usually in the form of ischemic colitis. We are reporting an 11-year-old Saudi girl with SCA who presented with persistent diarrhea and was found to have inflammaftory bowel disease.

## 1. Introduction

Homozygous sickle cell anemia (SCA) is an autosomal recessive chronic haemoglobinopathy characterized by abnormal globin chain of hemoglobin (Hb) content of the red blood cells that result in “sickle” shapes, attraction of RBCs to each other, and polymerization when in a low oxygen environment. The RBC polymerization leads to manifestations such as chronic occlusion of blood vessels (vasoocclusion), reduced blood flow to vital organs (ischemia), and alterations of the immune system. The gastrointestinal manifestation of SCA varies [1, 2]. We are reporting a child with SCA who presented with chronic colitis resembling inflammatory bowel disease.

## 2. The Case 

**Figure 1:**
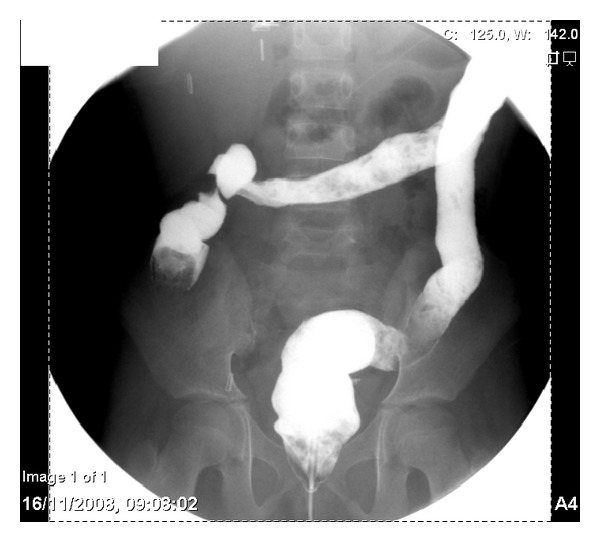
Gastrografin enema examination demonstrates loss of haustral folds in the colon with small ulcerations.

**Figure 2:**
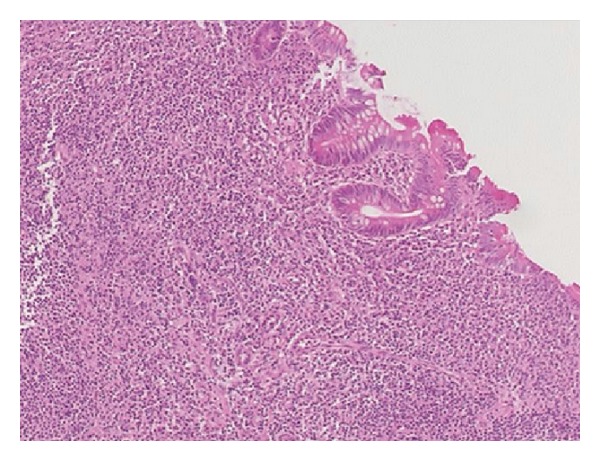
Ileal biopsy showed villus flattening with heavy infiltration of admixture of inflammatory cells rich in plasma cells.

**Figure 3:**
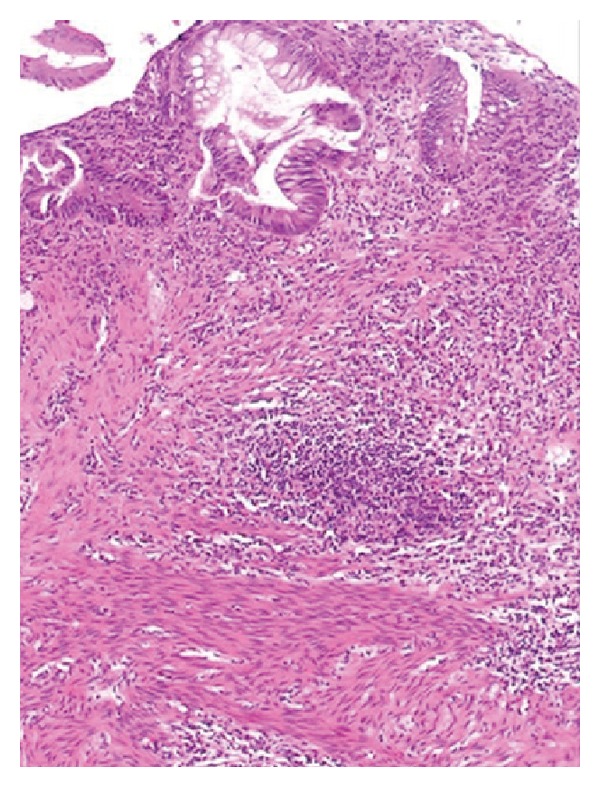
Colonic biopsy showed focally branched and distorted glands and heavy infiltration by lymphocytes, plasma cells, and eosinophils. The muscularis mucosa is thickened and inflamed.

An 11-year-old Black Saudi female with homozygous sickle cell (SS) disease (SCD) presented with chronic diarrhea for more than one-year duration. Her bowel motions were mostly watery but occasionally were mixed with little blood. It was occurring up to six to eight times per day, in moderate volume, and occasionally disturbed her sleep at night. This diarrhea was associated with poor appetite and poor weight gain. She also had a history of urgency and tenesmus most of the time. The patient had history of mild intermittent periumbilical colicky abdominal pain for same duration. She and her parents denied any history of vomiting, abdominal distension, recurrent or persistent fever, joint pain or swelling, skin rash, or mouth ulcers. The patient has a history of frequent admissions in the past due to vasooclusive and hemolytic crisis and received blood transfusion many times for this reason. Laparoscopic cholecystectomy was done at the age of eight years. She is the second child for young healthy consanguineous parents. One older brother died with sickle cell disease complicated by stroke. She has another two brothers and one sister all alive and well. On physical examination, she looked pale but not jaundiced. She has mild finger clubbing, normal skin examination, and no lymphadenopathy. Her weight was 18 kg and height was 127 cm, both far below the third percentile for her age. Her vital signs were all within normal limits. The perianal examination revealed no abnormalities. The rest of physical examination was unremarkable. Her initial laboratory workup showed white blood count of 19000 cells/cm^2^ (85% neutrophils and eosinophils 0%), hemoglobin 7.8 g/dL, and platelets 631000/mm^3^ with reticulocyte 10.7%. Her erythrocyte sedimentation rate (ESR) was 60 mm and C-reactive protein was 10 mg/dL. The serum albumin was low (26 g/L) and total protein was 63 g/L. All serum electrolytes, urea, and creatinine were within normal limits. Serum immunoglobulins (IgG, IgA, and IgM) were normal. Screening for celiac disease using serum antiendomysial antibodies test was negative. Liver enzymes and bilirubin were within normal limits. Several stool analyses and cultures were negative for virus, ova, or parasite and were negative for* Clostridium difficile *cytotoxin as well. The serology tests for human immunodeficiency virus were negative. Barium meal and follow through study revealed a mild irregularity with nodular hypertrophy of the terminal ileum while gastrografin enema (Figure 1) showed mucosal thickening of the entire colon with loss of haustral folds. The patient underwent upper endoscopic and colonoscopic examinations. Upper gastrointestinal endoscopy and histology were normal. Colonoscopy revealed nodular terminal ileum with otherwise normal mucosa. The entire colon was abnormal and had a picture of pancolitis in form of friable and edematous mucosa with diffuse erythema and decreased vascular markings. Few scattered pseudopolyps could be seen in the colon as well. The biopsies from terminal ileum (Figure 2) showed scattered hyperplastic lymphoid follicles with germinal centers. One fragment shows complete villus flattening with heavy infiltration of admixture of inflammatory cells rich in plasma cells. Colonic biopsies (Figure 3) showed focally ulcerated colonic mucosa replaced by fibroblastic and inflammatory granulation tissue. The lamina propria contained focally branched and distorted glands and heavy infiltration by lymphocytes, plasma cells, and eosinophils with the presence of scattered lymphoid follicles. The muscularis mucosa was thickened and inflamed. No granuloma could be seen at any level.

The patient was managed as an inflammatory bowel disease case (indeterminate type). She was started initially on oral sulfasalazine (1.5 g daily) but oral corticosteroid (prednisolone 40 mg daily) had to be added later on because of poor response. The patient started to improve and show signs of remission. She did well after that with no more diarrhea or abdominal pain and started to have better appetite. Her ESR went down to 5 mm and her serum albumin started to rise up. Prednisolone dose was tapered till discontinued. She was kept on the same dose of sulfasalazine as maintenance therapy. She was seen regularly in the clinic for more than six months with no significant relapse so far.

## 3. Discussion

SCA is a systemic disease that can affect many organs in the body including gastrointestinal tract. However, colonic involvement is very rare. Our patient represents a rare combination of SCA and chronic colitis. Review of the literature revealed only a single report of such association. Terry et al. [3] described four patients with SCA (one child) who presented with persistent diarrhea and chronic colitis. Those patients were diagnosed later on with ulcerative colitis based on clinical, radiological, and histopathological findings. The differential diagnosis of colitis in SCA patients should include ischemic and infectious colitis. In our patient, no organism could be isolated despite multiple cultures and examination of stool, blood, and urine. Furthermore, the histopathological and radiological findings were not supporting this possibility. Ischemic colitis had been reported in the literature emphasizing the liability of SCA patient to occlusion of blood vessels (vasoocclusion) and ischemia. However, ischemic colitis is not that common among sicklers and only eight cases were reported so far [2, 4–10]. This is likely because the colon has an abundant collateral blood supply and low oxygen extraction. The bowel can tolerate up to a 75% reduction of mesenteric blood flow for up to 12 hours with no ischemic changes [11].

The typical features of ischemic colitis include acute abdominal pain that persist despite conservative management associated with colonic changes in the form of segmental distribution of disease, abrupt transition between injured and uninjured mucosa, and rectal sparing. Colonic biopsies in several cases clearly demonstrated sickling within the vasculature of the diseased colonic segment [8].

Our patient presented with long standing chronic diarrhea which is unusual for ischemic colitis. In addition, the involvement of the entire colon that was seen on radiological, endoscopic, and histopathological evaluation makes ischemic colitis less likely. We do believe that the whole picture would be explained by inflammatory bowel disease especially after marked improvement with immunomodulation therapy. However, because inflammatory bowel disease is less common in children—particularly among Africans and Arabs—[12–14] and in addition to the complexity of SCA, further studies are needed to evaluate and explain more the relationship between these two disorders.
